# Changes in land use driven by urbanization impact nitrogen cycling and the microbial community composition in soils

**DOI:** 10.1038/srep44049

**Published:** 2017-03-10

**Authors:** Haitao Wang, Christopher W. Marshall, Minying Cheng, Huijuan Xu, Hu Li, Xiaoru Yang, Tianling Zheng

**Affiliations:** 1 Key Laboratory of the Ministry of Education for Coastal and Wetland Ecosystems, School of Life Sciences, Xiamen University, Xiamen 361102, China; 2 Key Lab of Urban Environment and Health, Institute of Urban Environment, Chinese Academy of Sciences, Xiamen 361021, China; 3 Biosciences Division, Argonne National Laboratory, Argonne, Illinois 60439, USA; 4 Department of Surgery, University of Chicago, Chicago, Illinois 60637, USA; 5 School of Architecture, South China University of Technology, Guangzhou 510641, China

## Abstract

Transition of populations from rural to urban living causes landscape changes and alters the functionality of soil ecosystems. It is unclear how this urbanization disturbs the microbial ecology of soils and how the disruption influences nitrogen cycling. In this study, microbial communities in turfgrass-grown soils from urban and suburban areas around Xiamen City were compared to microbial communities in the soils from rural farmlands. The potential N_2_O emissions, potential denitrification activity, and abundances of denitrifiers were higher in the rural farmland soils compared with the turfgrass soils. Ammonia oxidizing archaea (AOA) were more abundant than ammonia oxidizing bacteria (AOB) in turfgrass soils. Within turfgrass soils, the potential nitrification activities and AOA abundances were higher in the urban than in the suburban soils. These results indicate a more pivotal role of AOA in nitrification, especially in urban soils. Microbial community composition was distinctly grouped along urbanization categories (urban, suburban, and rural) classified according to the population density, which can in part be attributed to the differences in soil properties. These observed changes could potentially have a broader impact on soil nutrient availability and greenhouse gas emissions.

Urbanization has been increasing worldwide, with considerable growth in developing countries. The rapid transition of populations from rural to urban areas demands more resources and living spaces. As a result, large amounts of agricultural areas and forests have been replaced with urban and residential land use including impervious surfaces and managed green spaces^1^. Turfgrass ecosystems, dominating the urban green spaces, are created and maintained by humans for aesthetic and recreational purposes^2^ and are expected to expand at an unprecedented rate in the following decades^3^. Since these green spaces are the predominant means of carbon storage in urban areas^4^, inappropriate management can lead to environmental problems. For example, over fertilization of turfgrass can result in higher emissions of N_2_O^3^, a significant greenhouse gas with a 300-fold greater warming potential than CO_2_^5^. Many studies have investigated greenhouse gas emissions from urban soils^1^,^2^,^6^,^7^,^8^,^9^,^10^,^11^, however, information is limited on the soil microbiomes associated with biogeochemical cycles in urban green spaces.

Research on nitrogen cycling in urban areas have largely overlooked microbial ecology and instead focus on gas production and the associated environmental variables. Observations in Baltimore, Maryland demonstrated that N_2_O fluxes and nitrate leaching rates from urban lawns could be as high as the fluxes from forest plots in the same metropolitan area^2^,^12^. While urban lawns in Colorado occupied 6.4% of a study region, they contributed up to 30% of the regional N_2_O emissions^11^. The increased N_2_O emissions in urban lawns may be attributed to fertilization and increased temperature, either by climate change or the urban heat island effect or both^3^. Fertilizers and pesticides used for greenery establishment and maintenance also contribute to high N_2_O emissions^13^,^14^,^15^. While several studies looked at the variations of microbial community compositions in urban soils^8^,^16^,^17^, the functional communities that directly produce N_2_O remain under-investigated.

Nitrification and denitrification are the two main biogenic processes for N_2_O production in the soil^18^. While heterotrophic denitrification has been considered to be the most significant source of atmospheric N_2_O, nitrification is a major contributor under certain conditions^19^,^20^. For example, nitrification has been identified as the predominant contributor of N_2_O emitted from the wheat production system on the North China Plain where ammonium-based fertilizers were overused^21^,^22^. Urban turfgrass ecosystems are similar to wheat systems as they are both frequently fertilized, watered, and under oxic conditions. Therefore, it is hypothesized based on these similarities that the major metabolic route of N_2_O emissions from urban turfgrass soils will be through nitrification.

As the largest developing country, China has experienced both a rapid urbanization and an economic boom in the past 30 years^23^. The proportion of the urbanized population is expected to keep increasing over the next few decades^24^, thereby making the management of urban soils increasingly important for environmental and ecological health. To date, few studies have focused on the microbial communities in urban soils in China and little is known about how soil microbial communities respond to urbanization. To address this question, we selected Xiamen City as a test case because of the rapid urbanization of this area in recent years. In addition to soil properties, we investigated potential nitrification and denitrification activities, the corresponding nitrifying and denitrifying gene abundances, and overall microbial community composition in soils with different urbanization categories (urban/suburban/rural) classified according to the population density. Land use effect was compared between turfgrass and farmland soils while urbanization effect was compared among these categories. We hypothesized that land use and urbanization have a significant impact on microbial community composition and on nitrifying and denitrifying processes.

## Results

### Soil chemical properties

**Figure 1:**
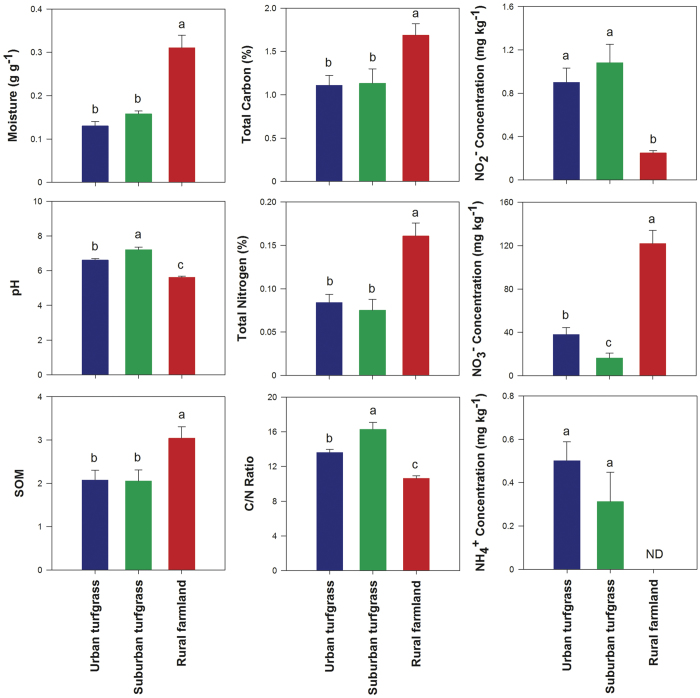
Soil chemical properties in urban (blue), suburban (green) and rural (red) categories. Error bars represent standard error of the mean (n = 32, n = 16 and n = 8 for urban, suburban and rural, respectively), and differences are significant when no same letter above the bars exists (*p* < 0.05). ND, not detected.

The distribution of sampling sites is displayed in Supplementary Fig. S1. These sites were classified into different categories according to the land-use type and population density (Table 1 and Supplementary Table S1). For convenience the groupings are referred to as urban and suburban turfgrass soils and rural farmland soils. Variations in the chemical properties of soil in different categories and each sampling site are displayed in Fig. 1 and Supplementary Table S2, respectively. The soil moisture, soil organic matter (SOM), total carbon (TC), total nitrogen (TN), and nitrate concentration were significantly higher in farmland soils compared to turfgrass soils, while pH, C/N ratio, nitrite, and ammonium concentrations were higher in the turfgrass (Fig. 1). Within the turfgrass grouping of samples, pH and C/N ratio were significantly higher and nitrate lower in suburban soils compared to urban soils (Fig. 1). Ammonium concentration was generally low in all the samples (below 1 mg NH_4_^+^ kg^−1^ dry soil), with some under detection limit (Supplementary Table S2). Nitrite concentration was low in all the samples while nitrate concentration was high in most of the samples, especially in farmland soils (Supplementary Table S2). Concentrations of other elements were highly variable between different sites in urban, suburban, and rural soils (Supplementary Table S3). Textures of soils from Urb1 were loamy sand while the soils from other sites were mostly silt loam, and a few were silt soil and sandy loam (Supplementary Table S4), suggesting the similarity of soil types among different sites.

### Potential microbial activities

**Figure 2:**
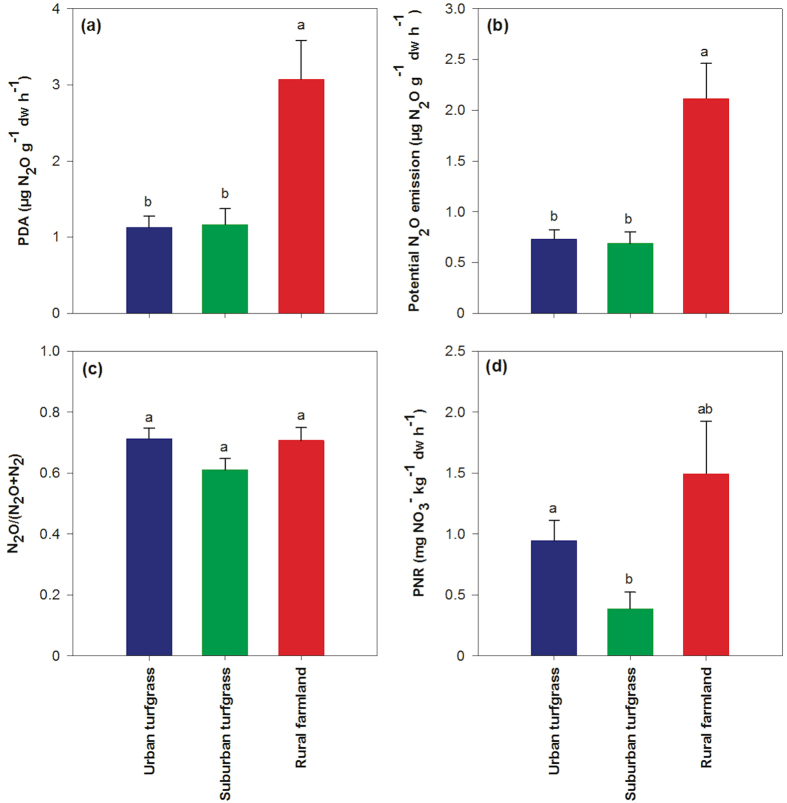
PDA (**a**), potential N_2_O emission (**b**), N_2_O/(N_2_O + N_2_) (**c**) and PNR (**d**) in urban (blue), suburban (green) and rural (red) categories. Error bars represent standard error of the mean (n = 32, n = 16 and n = 8 for urban, suburban and rural, respectively), and differences are significant when the letter above the bars are different (*p* < 0.05). PDA, potential denitrification activity; PNR, potential nitrification rate; N_2_O/(N_2_O + N_2_), potential N_2_O emission/PDA, the proportion of N_2_O emission to the total gas emission in denitrification.

Potential N_2_O emissions and potential denitrification activity (PDA) (2.62 and 4.10 μg N_2_O g^−1^ dw h^−1^, respectively) in paddy soils of the Rur2 site were the highest among all the sampling sites (Supplementary Table S5). As a result, the overall potential N_2_O and PDA were significantly higher in the rural farmland soils compared with the turfgrass soils (Fig. 2a and b). However, the N_2_O/(N_2_O + N_2_) ratio, the proportion of N_2_O emission to the total gas emission in denitrification, showed no variation among these three categories (Fig. 2c). There was no difference in potential nitrification rate (PNR) between rural farmland and turfgrass soils whereas urban soils exhibited a significantly higher PNR compared with suburban soils (Fig. 2d). Overall, the potential microbial activities were significantly different along the urbanization gradient groupings with the exception of N_2_O/(N_2_O + N_2_) (Table 2). Correlation analysis demonstrated that nitrate concentration was significantly and positively correlated with potential N_2_O emission (R = 0.652, *p* < 0.001), PDA (R = 0.703, *p* < 0.001) and PNR (R = 0.625, *p* < 0.001).

### Gene abundances

The abundances of genes involved in nitrification and denitrification were all significantly higher in the rural farmland than in the turfgrass soils, with the exception of ammonia oxidizing archaea (AOA) (Table 3). Within the turfgrass groupings, the AOA abundance was higher in the urban soils compared to the suburban soils (Table 3). The ratio of AOA to ammonia oxidizing bacteria (AOB) showed that AOA was more abundant than AOB in all sites with the exception of Rur2, where AOA and AOB were equally abundant (Supplementary Table S6). According to the *nirK/nirS* ratio, *nirS* denitrifiers were predominant over *nirK* in rural farmland soils whereas these were equivalent in urban and suburban turfgrass soils. Denitrifiers with *nosZ* clade II outnumbered *nosZ* clade I denitrifiers in most soils, indicating the predominant role of clade II over *nosZ* I in these soils (Supplementary Table S6). The *nir*/*nos* ratio was lower in the urban turfgrass soils compared with the other two groups (Table 3). The relative abundance was calculated as abundance ratio of each functional gene to corresponding 16 S rRNA gene, as the 16 S rRNA gene quantification showed that total bacteria and archaea were unequal in soils from different sites (Supplementary Table S6). Results revealed that the relative abundance of AOB, *nirS,* and *nosZ* I in urban turfgrass sites were significantly lower than in the suburban turfgrass and rural farmland sites (Table 3). However, AOA relative abundance was greater in urban sites compared with the rural sites (Table 3). Kruskal-Wallis H test suggested that urbanization greatly influenced the abundances of nitrifiers and denitrifiers, despite observed exceptions (Table 2).

### Diversity and composition of microbial communities

**Figure 3:**
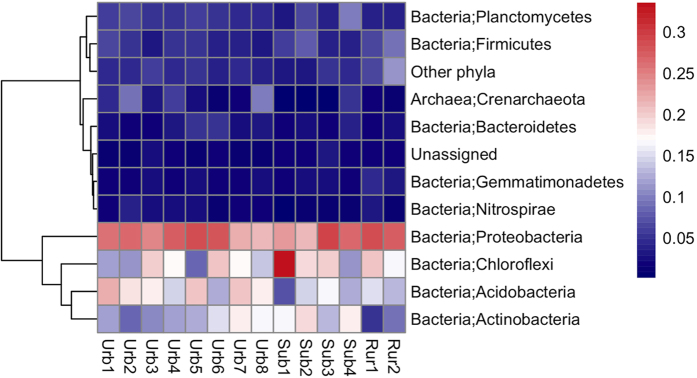
Heatmap showing average relative abundances of microbial communities at phylum level. The clustering of taxa is based on the Pearson’s correlation.

The alpha-diversity was higher in the rural farmland soils than in the urban and suburban turfgrass soils based on the phylogenetic diversity (PD) and Shannon index (Supplementary Fig. S2). The most abundant phylum was Proteobacteria, accounting for 20.9–29.1% relative abundance in different sites, followed by Chloroflexi, Acidobacteria, and Actinobacteria (accounting for 8.8–33.6%, 7.5–22.2% and 5.3–19.1%, respectively) (Fig. 3). Acidobacteria were more abundant in the urban soils than in the suburban and rural agricultural soils while Actinobacteria relative abundance was higher in the suburban soils compared to urban and rural agricultural soils (Fig. 3). Within the Proteobacteria, the Alphaproteobacteria, Betaproteobacteria, Deltaproteobacteria, and Gammaproteobacteria represented 8.6%, 5.0%, 8.6% and 3.3% of the total bacteria across all sites, respectively. Only 2.6% of sequences were assigned to archaea, but this is common in soil microbial communities despite our bacterial specific primers. To evaluate the accuracy of the probes, probe match from the Ribosomal Database Project was conducted^25^. Results showed that of the 154,357 total archaea taxa, there were 631 hits and 92,043 hits when nucleotide difference allowed was 1. This suggests that the occurring of archaeal sequences was not due to operating contamination.

**Figure 4:**
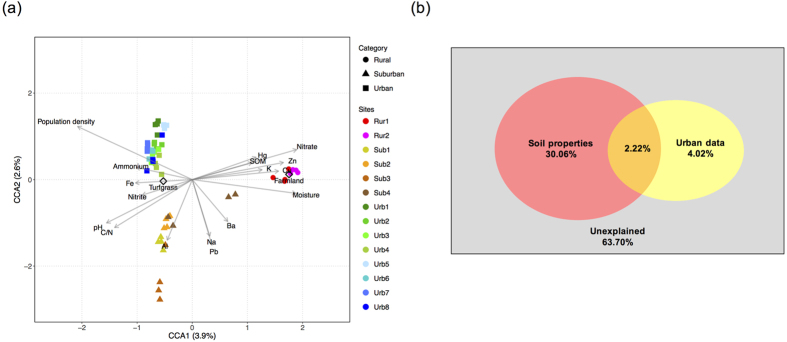
(**a**) Canonical correspondence analysis (CCA) based on the relative abundance of each OTU. 16 significant soil properties and 2 urban data were selected as the environmental variables. The quantitative variables are represented by arrows and the categorical variable, land use (“Turfgrass” and “Farmland”), is shown as unfilled diamonds. (**b**) Variation partitioning analysis for the explanatory proportions of different sets of factors. The land use was transformed to dummy variables for this analysis (turfgrass, 1; farmland, 0).

Principal coordinate analysis (PCoA) plots showed that microbial communities from different sites were distinctly grouped according to the three urbanization categories with the exception of two samples from Sub2 (Supplementary Fig. S3a). Analysis of similarities (ANOSIM) and permutational multivariate analysis of variance (PERMANOVA) revealed that the microbial community compositions from urban, suburban and rural soils were significantly different from each other based on both unweighted and weighted Unifrac matrices (Supplementary Table S7). Similarly, canonical correspondence analysis (CCA) exhibited distinct groupings along the urbanization category (Fig. 4a). Permutation tests revealed that both the overall test (*p* = 0.001) and first two axes (*p* = 0.001 and *p* = 0.001) of the CCA were significant. Fe, ammonium, and population density were more positively correlated with microbial communities from urban soils while pH, C/N, and Al correlated with suburban microbial communities. Hg, K, Cu, Zn, SOM, nitrate and moisture showed stronger positive relationships with microbes in the rural agricultural soils. Fitting these variables into PCoA plots showed the similar results (Supplementary Fig. S3). All these 18 factors explained 36.30% of the microbial community variance (Fig. 4b). As population density was represented by the urbanization categories in this study, it was classified as the urban data along with land use, while the other factors corresponded to soil properties. The soil properties could explain 30.06% of the variance while urban data only explained 4.02% (Fig. 4b). The interaction between the two factor sets explained 2.22% (Fig. 4b). Among these factors, pH, C/N and nitrate along with land use and population density exhibited the strongest effects on the microbial community composition (Fig. 4a). This is also supported by the Mantel test showing significant correlations between community composition and these factors (Supplementary Table S8). There are three abundant genera, *Candidatus Nitrososphaera, Nitrospira* and *Nocardioides*, which accounted for 2.3%, 0.8% and 0.3% of the total sequences respectively, showed significant correlations with both weighted and unweighted PCoA1 (*p* < 0.05), suggesting their pivotal roles in shaping the community structures.

**Figure 5:**
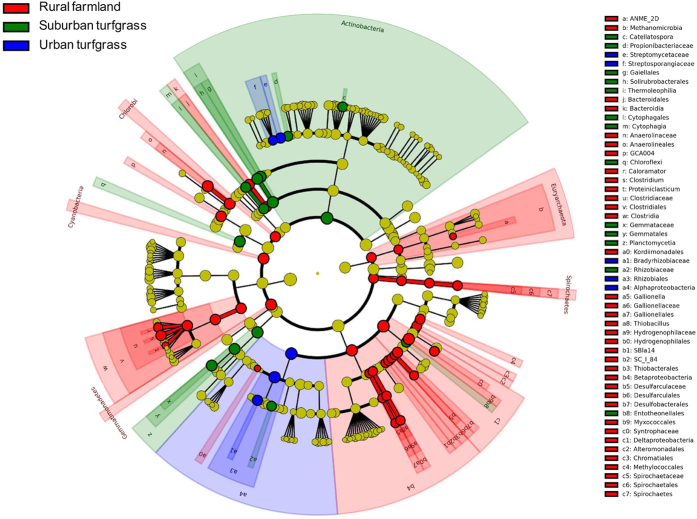
Non-strict version of LEfSe results on microbial communities. The cladogram indicates the taxa (highlighted with small circles and shading) showing different abundance values (according to LEfSe) in the urban, suburban, and rural agricultural soils. For each taxon (circle), the color denotes the significantly higher abundance of the taxon in the corresponding group. Yellow denotes that the taxon is not significantly higher in any group.

We used LDA Effect Size (LEfSe)^26^ to determine the significant taxa in each category (urban, suburban, and rural). The most differentially abundant microbial taxa in urban soils belonged to the Rhizobiales order and Bradyrhizobiaceae family in the class Alphaproteobacteria, and two families, Streptomycetaceae and Streptosporangiaceae, from the Actinobacteria phylum (Fig. 5 and Supplementary Fig. S4). In the suburban soils, orders Gaiellales, Solirubrobacterales, and class Thermoleophilia in the Actinobacteria phylum and members in the order Chloroflexi were the most differentially abundant taxa in suburban soils (Fig. 5 and Supplementary Fig. S4). Of the many significantly different taxa in the rural sites, the most significant taxa included the class Deltaproteobacteria, the order Anaerolineales, the phylum Cyanobacteria, and the order GCA004 (Fig. 5 and Supplementary Fig. S4).

## Discussion

Nitrogen cycling and microbial community structure in soils with different land uses and urbanization categories were investigated in the present study. The results demonstrated significant differences in potential activities and abundances of microbial communities involved in nitrogen cycling. Additionally, microbial community composition and structure differed between turfgrass sites and rural farmland sites and within turfgrass sites between suburban and urban sites. These findings support the hypotheses that land use changes along with urbanization influence the microbial processes involved in nitrogen cycling and the overall microbial community composition in soils.

Conversion of land use contributes to shifts in ecosystem processes by shaping the soil microbial community diversity and function^27^. This study focused on the changing of rural farmlands into high population urban areas. Higher potential N_2_O emission and denitrification activity was observed in rural agricultural soils compared to urban and suburban turfgrass soils, suggesting that this land use change as a result of urbanization may reduce N_2_O emissions from denitrification. Similar patterns were observed in the *nirK* and *nirS* gene abundances and the lower *nir*/*nos* in urban turfgrass soils suggest more compete denitrification and less N_2_O emission, lending evidence to support potential activity as a proxy for field activity and the reduction of N_2_O as a result of urbanization. This would not typically be surprising since the rural agricultural lands normally receive more nitrogen input and watering which create the optimal environment for denitrifying bacteria. However, it is a different situation in urban turfgrass soils. The lower moisture content allows more oxygen, which can stimulate nitrification. While ammonium concentrations were low or under detection limit in this study, high nitrate concentrations were observed in most of the turfgrass soils (over 20 mg NO_3_^−^ kg^−1^, Supplementary Table S2). These concentrations were much higher compared with those from home lawns, forests, urban grasslands, and agricultural lands in the United States^2^,^28^, suggesting the heavy use of fertilizers in the management of turfgrass in China. Considering that ammonium-based fertilizers are the most commonly used, the low ammonium concentrations, and a significant correlation between PNR and nitrate concentration, we infer that nitrification is probably predominant over denitrification, thus primarily contributing to N_2_O emission in urban turfgrass soils. This speculation is consistent with studies from crop-based systems that use ammonium-based fertilizers^21^,^22^. Hence, possibility that the field emission of N_2_O in turfgrass soils might be comparable to that in agricultural soils exists, which warrants further studies to verify.

Within the turfgrass sites, we found that the PNR and AOA abundance were higher in urban soils than that in suburban soils. Additionally, AOA were significantly more abundant compared to AOB and were thus the major contributors to nitrification in turfgrass soils. These results suggest the dominant role of AOA over AOB on nitrifying process in turfgrass soils, with more significance in urban soils.

The microorganisms containing the *nosZ* clade II gene have been identified as primarily responsible for reducing N_2_O emissions in different ecosystems because they lack the preceding steps in the denitrification pathway^29^,^30^,^31^. However, the distribution and ecology of this community remains unclear. Notably, we found that *nosZ* clade II was in higher abundance than *nosZ* clade I in most of the sampled soils, suggesting that *nosZ* II denitrifiers are of great significance in urban turfgrass soils. Given the importance of *nosZ* II communities, more work is required to reveal the pattern of their community compositions in different urban ecosystems.

Biodiversity is an important indicator of ecosystem service and greater biodiversity generally corresponds to proper ecosystem functioning, thereby raising concerns about the consequences of biodiversity decline^32^,^33^,^34^. In this study, the diversity in turfgrass soils was significantly lower than in the rural farmland soils, suggesting that the conversion of land use driven by urbanization may cause a decline in biodiversity and potentially imperil ecosystem function. For instance, it is suggested that reduced exposure to microorganisms results in respiratory disease^35^ and defective regulation of the immune system^36^. As urbanization is forcing humans to limit their exposure to rural environments, the loss of microbial diversity might lead to both allergic diseases and public health problems in general^37^.

The structure of microbial communities is of great importance to ecosystem functioning as microorganisms govern biogeochemical cycling and are more diverse than any other organisms^38^. We found a pattern of microbial communities that significantly grouped along the urbanization categories and were correlated to soil physicochemical properties. The nitrate and pH showed the most significant influences on the microbial community since the rural soils received more fertilizers which changed the soil pH. The land use and population density were correlated with the distribution of the urbanization categories and therefore showed a strong effect on the community. The significant differences between each two of the microbial communities in urban, suburban and rural soils also suggest that the land use could shape the microbial community and the influence is associated with urbanization.

The majority of the microbial community composition remained unexplained by the variables measured in this study. However, there are many factors shaping the communities in the soils in addition to physicochemical properties. For instance, habitat patches in the city are subject to varying degrees of environmental stress, including habitat fragmentation and increased heat^39^,^40^, which leads to further changes in soil properties. A recent study also showed that urban stress like habitat patchiness significantly shaped bacterial community compositions^41^. Moreover, it is identified that geographic and urbanization indices are significant predicting factors for bacterial community composition in urban park soils^16^. As urbanization is accompanied with environmental changes at multiple scales, future studies on the interaction between urbanization and biological variations should include influencing factors at different scales or stresses at different degrees.

An archaeal genus in the Thaumarchaeota phylum, *Candidatus Nitrososphaera,* was abundant and correlated with the first principal coordinate based on weighted unifrac distance of community diversity in this study. This genus is an important cluster of AOA, with only two species currently recognized^42^,^43^. The other microbial genus correlated to community structure was *Nitrospira,* which possesses the capabilities to perform complete nitrification^44^. While *Candidatus Nitrososphaera* was more abundant in urban soils (4.4%) compared with suburban (1.6%) and rural (0.8%) soils, *Nitrospira* showed a higher abundance in rural soils (1.4%) than in urban (0.9%) and suburban (0.2%) soils. These results suggested that AOA and AOB were important microbial guilds for the variability of the microbial communities, with different significances in urban and rural soils.

As land-use types were different between turfgrass soils and rural agricultural soils, the abundant taxa in urban and suburban soils were distinguishable from those in rural soils. Acidobacteria and Actinobacteria were enriched in urban and suburban soils. They are both widely distributed bacteria in soils and are thought to be important contributors to ecosystems^45^,^46^. Additionally, nitrogen-fixing taxa (Rhizobiales and Rhizobiaceae) were found to be abundant in turfgrass soils, suggesting an increased demand for symbiotic bacteria in turfgrass. Proteobacteria and Chloroflexi were abundant in rural soils, including many sub-taxa with capabilities of nitrification, denitrification, sulfate reduction, methane oxidation, and iron reduction/oxidation. These processes normally occur in oxic-anoxic transition zones such as the rhizosphere in flooded paddy soils^47^. The distinct distribution of microbes in turfgrass and farmland soils illustrates that conversions of land use and land cover accompanying urbanization may heavily alter the microbial communities, thus disturbing biogeochemical cycling.

Our results indicate that urban turfgrass soils are hot spots for nitrogen cycling and that changes associated with urbanization can significantly impact the nitrogen cycling. The higher PDA and denitrifying gene abundances in farmland soils compared to turfgrass soils indicate that land use changes by urbanization may negatively contribute to denitrifying processes. We also found the predominant role of AOA over AOB in turfgrass soils and that AOA were more significant in urban soils. The reduction of microbial diversity in urban and suburban soils could also result in the decline of ecosystem functionalities. The distinct community composition between groupings suggests the influence of urbanization on potential soil functions. Overall, the present study shows that land use changes driven by urbanization can significantly impact nitrogen cycling and shape microbial communities. Despite this progress, the full impact of urbanization on soil microbial communities, biogeochemical cycling, and greenhouse gas emissions remains unclear and warrants further investigation in different urban ecosystems around the world.

## Methods

### Study area and soil sampling

The study area was in Xiamen, a coastal city in Southeast China (17°53′-118°25′E and 24°25′-24°54′N). It experiences a subtropical marine climate with an annual average temperature of 21 °C. The annual average precipitation is approximately 1200 mm and the relative humidity is 76%. This city is one of the earliest Special Economic Zones (SEZs) established by Chinese government in 1980. Since then, Xiamen has experienced a rapid urbanization and an economic boom, leading to significant changes in ecosystem services and landscape patterns^48^. The proportion of farmland areas had been reduced from 37.7% in 1987 to 19.5% in 2012, while the developed areas have increased from 3.5% in 1987 to 23.4% in 2012 (Supplementary Fig. S1). Additionally, the overall population of Xiamen City increased from 2.05 million in 2000 to 3.81 million in 2014^49^. For this study, we classified these three districts based on their urbanized degrees (population densities) according to Ren *et al*.^50^. Siming and Huli are classified as urban areas with a population over 10 thousand per km^2^, while Jimei is recognized as the suburban area with only around 2.4 thousand people per km^2^ (Supplementary Table S1)^49^. The population densities of sub-districts also showed the significant pattern according to urban and suburban categories (Table 1).

In this study, eight representative sites in urban areas, four sites in suburban areas, and two sites on the outskirts of Jimei were chosen as study sites (Table 1, Supplementary Fig. S1). Among the urban and suburban sites, one site was botany garden and Meihailing Park was located inside a forest near the built up area and it was frequently visited by people since it was a City Park. However, all the lawns selected in these sites were planted with similar grass species. The two rural sites were vegetable and paddy fields. The four sites in Jimei and two sites in Huli were used as the farmland in 1987 and then they were altered to build up area by urbanization (Supplementary Fig. S1). Two farmlands from the Jimei rural area remained unchanged since 1987 (Supplementary Fig. S1) and they represent the historical state of soils currently classified as urban and suburban landscapes. These sites were compared to investigate the influence of land use change.

All soil samples were collected on September 3rd and 5th, 2014. At each site, four replicates were set by collecting samples from four locations with a distance of around 10 m. There were 56 total samples collected (14 sites x 4 locations). Soils were sampled at 0–20 cm, immediately blended and transported to the laboratory on ice for further analysis. Samples used for molecular analyses were stored at −80 **°**C.

### Soil properties and potential activities

Soil moisture content was gravimetrically measured by drying the soil for 12 h at 105 °C. pH was determined by using a XL60 pH meter (Fisher Scientific, USA) in the solution (1:2.5 grams of dry soil:mL of water). TC and TN were measured with vario MAX CNS elemental analyzer (ELEMENTAR, German). Ammonium (NH_4_^+^), nitrate (NO_3_^−^), and nitrite (NO_2_^−^) were extracted with distilled water (1:5 grams of soil:mL of water) and analyzed using an ICS-3000 ion chromatography (Dionex, USA) as previously described^51^. Soil texture was measured using a MS2000 laser particle size analyzer (Malvern, UK). SOM was calculated by total organic carbon (TOC) multiplying factor 1.724. TOC was measured by a TOC-Vcph analyzer (Shimadzu, Japan). Dried soils were digested with HNO_3_ and HClO_4_. Elements (K, Ca, Na, Mg, Cu, Mn, P, Zn, Cr, Ba, Fe, Al, Ni, As, Hg and Pb) were measured by analyzing the digested solutions with Optima 7000DV ICP-OES (PerkinElmer, USA) and 7500cx ICP-MS (Agilent, USA).

PDA and potential N_2_O emission were measured as previously described with some modifications^52^ (Supplemental Methods). The collected gases were analyzed using a gas chromatographer equipped with an ECD detector as previously described^53^. PDA is expressed as N_2_O + N_2_ while the potential N_2_O emissions are simply referred to as N_2_O. PNR was measured as accumulated nitrate in a short incubation modified from previous study^54^ (Supplemental Methods).

### DNA extraction and gene quantification

Total DNA was extracted from 0.5 g fresh soil using the FastDNA SPIN Kit for Soil (MP Biomedicals, Santa Ana, CA, USA) according to the manufacturer’s instructions. The extracted DNA was then purified by a PowerClean DNA Clean-Up Kit (Mo Bio Laboratories, CA, USA). DNA concentrations were measured via spectrophotometer ND-1000 (NanoDrop, USA).

The real-time PCR assay was conducted in a 20 μL reaction mixture containing 20 ng of DNA, 0.2 μM of each primer, 4 μg of BSA (Takara, Japan) and 10 μL of 2 × SYBR Premix Ex Taq II (Takara). Primers and conditions used for quantifying denitrifiers (*nirK, nirS* and *nosZ* I) and nitrifiers (archaeal and bacterial *amoA*) were described in previous studies with modifications^55^,^56^ (Supplementary Table S9). The primers, nosZ-II-F/nosZ-II-R, 515 F/907 R and arch21F/arch958R were used to target the *nosZ* clade II communities, bacteria and archaea 16 S rRNA genes, respectively (Supplementary Table S9). PCR conditions of each gene were displayed in Supplementary Table S9. All reactions were conducted on a LightCycler 480II Real-Time PCR System (Roche, IN, USA). Standard curves were obtained using gradient dilutions of standard plasmids containing the target genes with known copy numbers. Negative controls without DNA template were included in each amplification. Inhibitors were eliminated in the purification step. PCR efficiency above 90% was accepted.

### Sequencing of the 16 S rRNA gene and data analysis

The V4 and V5 regions of the 16 S rRNA gene were amplified with 515 F/907 R primers^57^. The reverse primers (907 R) were tagged with a six-base barcode (Supplementary Table S10). The procedures to process the sequencing were displayed in supporting methods (Supplemental Methods).

Sequence data were analyzed using QIIME version 1.9.1^58^. Raw sequences were demultiplexed and low quality or ambiguous reads were removed and then chimeric reads were removed and checked. Filtered sequences were clustered into operational taxonomic units (OTUs) at 97% similarity level and the representative sequence for each OTU was selected. The representative sequences were then assigned to taxonomy using RDP^59^. OTUs with a single sequence or assigned to mitochondria or chloroplast were filtered. Samples were then rarefied to determine the alpha diversity and beta diversity (Supplemental Methods). LEfSe was calculated to identify the corresponding taxa with higher abundance in different samples (Supplemental Methods).

All the sequences were submitted to the European Nucleotide Archive of EMBL. The accession number is PRJEB14752.

### Statistical analysis

Considering the unbalanced sample size in three categories, the nonparametric Kruskal-Wallis H test was employed to observe the significance of urbanization impact on the potential activities and gene abundances. To compare the significance of variation between each two of categories, post hoc multiple comparisons were conducted using the Dunnett’s T3 method. Fisher’s Least Significant Difference (LSD) was calculated as previously described^60^, to compare the significance between each two sites. The counts in OTU table were transformed to relative abundances as percentage. CCA was then performed to determine the significant factors shaping the bacterial communities. Variation partitioning was used to reveal the proportions of variance explained by different factors by running the partial CCA (Supplemental Methods). A heatmap was generated to exhibit the relative abundance of phylum taxa in different samples. Pearson’s correlation analysis was conducted between each pair of the parameters or values measured in this study. ANOSIM and PERMANOVA were employed to test the significance of urbanization (urban, suburban and rural) impact on the beta-diversity based on the Unifrac matrices. Mantel tests were performed to explore the correlations between different variables and microbial communities based on the Unifrac matrices. Kruskal-Wallis H test, multiple comparisons and correlation analysis were carried out with SPSS v19.0 software (IBM, USA). The other analyses were performed using R v3.3.2 software (R Foundation for Statistical Computing, Vienna, Austria) (Supplemental Methods). The null hypothesis was rejected when *p*-values were less than 0.05.

## Additional Information

**How to cite this article:** Wang, H. *et al*. Changes in land use driven by urbanization impact nitrogen cycling and the microbial community composition in soils. *Sci. Rep.* **7**, 44049; doi: 10.1038/srep44049 (2017).

**Publisher's note:** Springer Nature remains neutral with regard to jurisdictional claims in published maps and institutional affiliations.

## Supplementary Material

Supplementary Information

## Figures and Tables

**Table 1:** Information and statistic data of each sampling site.

Site	Coordinate (N)	Coordinate (E)	District	Sub-district			Density(10^3^km^−2^)	Land cover	Category	Abbreviation
				Name	Area (km^2^)	Population(10^3^)				
Bailuzhou Park	24.47746	118.08905	Siming	Yuandang	9.5	225	23.68	turfgrass	urban	Urb1
Zhongshan Park	24.46098	118.08515	Siming	Kaiyuan	5.7	85	21.37	turfgrass	urban	Urb2
Botany Garden	24.45525	118.09002	Siming	Kaiyuan	5.7	85	21.37	turfgrass	urban	Urb3
Jinbang Park	24.46629	118.10471	Siming	Wucun	6.2	129	20.74	turfgrass	urban	Urb4
Meihailing Park	24.45295	118.12298	Siming	Wucun	6.2	129	20.74	turfgrass	urban	Urb5
Buzheng Park	24.48126	118.12530	Siming	Jialian	4.5	125	27.90	turfgrass	urban	Urb6
Zhonglun Park	24.48710	118.14848	Huli	Jiangtou	10.4	223	23.68	turfgrass	urban	Urb7
Jiangtou Park	24.49740	118.12496	Huli	Jiangtou	10.4	223	23.68	turfgrass	urban	Urb8
Lehai Park	24.57917	118.10732	Jimei	Jimei	3.6	43	11.94	turfgrass	suburban	Sub1
Jingxian Park	24.57744	118.09997	Jimei	Jimei	3.6	43	11.94	turfgrass	suburban	Sub2
Ridong Park	24.56477	118.03159	Jimei	Xingbin	21.6	190	8.80	turfgrass	suburban	Sub3
Xingdong Park	24.56504	118.04491	Jimei	Xinglin	24.7	122	4.93	turfgrass	suburban	Sub4
Houxi Vegetable Field	24.64128	118.03666	Jimei	Houxi	44.1	59	1.33	vegetable	rural	Rur1
Houxi Paddy Soil	24.64119	118.03547	Jimei	Houxi	44.1	59	1.33	rice	rural	Rur2

The population is the permanent residential population.

**Table 2:** Kruskal-Wallis H test for the effect of urbanization on the potential microbial activities and gene abundances.

	Chi square	Significance	Significant level
Potential N_2_O emission	13.35	0.001	***
PDA	15.35	<0.001	***
N_2_O/(N_2_O + N_2_)	3.98	0.137	NS
PNR	12.50	0.002	**
Bacterial 16 S abun.	16.17	<0.001	***
Archaeal 16 S abun.	15.07	0.001	***
AOA *amoA* abun.	28.74	<0.001	***
AOB *amoA* abun.	19.60	<0.001	***
AOA/AOB	23.73	<0.001	***
*nirK* abun.	13.35	<0.001	***
*nirS* abun.	25.57	<0.001	***
*nirK/nirS*	20.42	<0.001	***
*nosZ* I abun.	20.39	<0.001	***
*nosZ* II abun.	10.86	0.004	**
*nosZ* I*/nosZ* II	6.23	0.044	*
*nir/nos*	13.54	0.001	***
AOA *amoA* R.abun.	25.73	<0.001	***
AOB *amoA* R.abun.	10.45	<0.001	***
*nirK* R.abun.	4.05	0.132	NS
*nirS* R.abun.	17.83	<0.001	***
*nosZ* I R.abun.	16.88	<0.001	***
*nosZ* II R.abun.	0.48	0.788	NS

Urbanization refers to three categories (urban, suburban, and rural) defined by population densities. NS, not significant. abun., abundance. R. abun., relative abundance. *, significance level at 0.05; **, significance level at 0.01; ***, significance level at 0.001.

**Table 3:** Absolute abundances (copies g^−1^ dry weight soil), ratios and relative abundances of genes involved in nitrification and denitrification in soils with different categories.

	Urban turfgrass	Suburban turfgrass	Rural farmland
Archaeal 16 S rRNA (10^9^)	0.50 ± 0.05^b^	0.66 ± 0.25^ab^	1.29 ± 0.14^a^
Bacterial 16 S rRNA (10^10^)	1.34 ± 0.13^b^	1.30 ± 0.24^b^	3.37 ± 0.34^a^
AOA (10^8^)	1.86 ± 0.22^a^	0.28 ± 0.09^b^	0.41 ± 0.04^b^
AOB (10^7^)	0.30 ± 0.06^b^	0.89 ± 0.04^b^	3.10 ± 0.83^a^
*nirK* (10^8^)	1.91 ± 0.19^b^	1.98 ± 0.40^b^	4.06 ± 0.37^a^
*nirS* (10^9^)	0.22 ± 0.04^b^	0.55 ± 0.13^b^	2.16 ± 0.27^a^
*nosZ* I (10^8^)	0.26 ± 0.03^b^	0.36 ± 0.07^b^	1.16 ± 0.08^a^
*nosZ* II (10^8^)	1.06 ± 0.20^ab^	0.59 ± 0.13^b^	1.76 ± 0.24^a^
AOA/AOB	135.64 ± 26.41^a^	58.73 ± 19.97^ab^	2.08 ± 0.53^b^
*nirK*/*nirS*	1.92 ± 0.31^a^	1.11 ± 0.43^ab^	0.20 ± 0.02^b^
*nosZ* I/*nosZ* II	0.45 ± 0.05^b^	1.11 ± 0.25^a^	0.73 ± 0.09^a^
*nir*/nos*	4.80 ± 0.74^b^	9.71 ± 1.62^a^	9.62 ± 1.37^a^
AOA/16 S**	0.45 ± 0.05^a^	0.36 ± 0.24^ab^	0.03 ± 0.01^b^
AOB/16 S (10^−4^)	2.19 ± 0.32^b^	3.79 ± 1.66^ab^	9.46 ± 2.28^a^
*nirK*/16 S (10^−2^)	1.51 ± 0.11^a^	1.46 ± 0.06^a^	1.37 ± 0.30^a^
*nirS*/16 S (10^−2^)	1.75 ± 0.36^b^	6.80 ± 1.55^a^	7.07 ± 1.06^a^
*nosZ* I/16 S (10^−3^)	1.97 ± 0.12^b^	3.49 ± 0.58^a^	3.73 ± 0.47^a^
*nosZ* II/16 S (10^−3^)	7.97 ± 1.32^a^	5.80 ± 1.01^a^	5.82 ± 1.22^a^

Values are given as mean ± standard deviation (n = 32, 16, 8 for Urban, Suburban and rural, respectively). *nir/*nos*, (*nirK* + *nirS*)/(*nosZ* I + *nosZ* II). **16 S, archaeal 16 S rRNA for AOA and bacterial 16 S rRNA for the others. Differences are significant when no same letter exists between categories (*p* < 0.05).
