# Supplementary material for: Changes in land use driven by urbanization impact nitrogen cycling and the microbial community composition in soils
Source: Sci Rep. 2017 Mar 10;7:44049. doi: 10.1038/srep44049 (PMC5345093; doi:10.1038/srep44049)
Supplement: Supplementary Information [file srep44049-s1.pdf]

Changes in land use driven by urbanization impact nitrogen cycling and the microbial community composition in soils

Haitao Wang[1,2], Christopher W. Marshall[3,4], Minying Cheng[5], Huijuan Xu[2], Hu Li[2], Xiaoru Yang[2,*], Tianling Zheng[1,*]

[1]Key Laboratory of the Ministry of Education for Coastal and Wetland Ecosystems, School of Life Sciences, Xiamen University, Xiamen 361102, China
[2]Institute of Urban Environment, Chinese Academy of Sciences, Xiamen 361021, China
[3]Biosciences Division, Argonne National Laboratory, Argonne, Illinois 60439
[4]Department of Surgery, University of Chicago, Chicago, Illinois 60637
[5]School of Architecture, South China University of Technology, Guangzhou 510641, China
*Corresponding. T.Z., wshwzh@xmu.edu.cn, X.Y., xryang@iue.ac.cn

This PDF includes:

Supplementary Methods.
Supplementary Figure S1. Changes of land uses and the distribution of sampling sites.
Supplementary Figure S2. Rarefaction curves of the microbial communities.
Supplementary Figure S3. PCoA plots of the microbial communities.
Supplementary Figure S4. Histogram of the LDA scores.
Supplementary Table S1. The statistic data of Siming, Huli and Jimei in Xiamen in 2010, 2012 and 2014.
Supplementary Table S2. Soil chemical properties in each site.
Supplementary Table S3. Concentrations of elements in different sampling sites.
Supplementary Table S4. Soil texture of each soil in different sampling sites.
Supplementary Table S5. Potential N_2O emission, denitrification activities and nitrification rate in different sampling sites.
Supplementary Table S6. Absolute abundances, ratios and relative abundances of genes involved in nitrification and denitrification in different samples.
Supplementary Table S7. Pairwise ANOSIM and PERMANOVA between urban, suburban and rural based on Unifrac distances.
Supplementary Table S8. Mantel tests between environmental variables and microbial community based on Unifrac distances.
Supplementary Table S9. Primers and conditions used to target and quantify the 16S rRNA and functional genes.
Supplementary Table S10. Primers used to amplify V4 and V5 regions of 16S rRNA gene.

Methods

Potential activities

Potential denitrification activity (PDA) and potential N_2O emission were measured using an incubation method. Briefly, 10 g fresh soil was placed in a 120-mL serum bottle containing 10 mL solution of 0.4 mM glucose and 0.4 mM KNO_3, which was then evacuated and flushed with 99.999% helium three times. C_2H_2 was injected to a final composition of 10 % (vol/vol) to test for PDA. The sediments were then incubated at 25°C on a magnetic stirring apparatus. The headspace samples were analyzed at 2, 4 and 6 h using a gas chromatographer equipped with an ECD detector as previously described.[31] Activities were calculated by linear regression of accumulated N_2O per gram dry soil versus time. Potential nitrification rate (PNR) was measured as accumulated nitrate in a short incubation. Briefly, 15 g fresh soil was added to 100 mL ammonium sulfate solution (1.5 mM) which was then incubated at 25°C for 24 h on a rotary shaker at 180 rpm. During the incubation, aliquots of 10 mL subsamples were collected at 2, 8, and 24 h after the start of the incubation, which were then analyzed by an ICS-3000 ion chromatographer (Dionex, USA). The rates were calculated by linear regression of the accumulated NO_3^- per gram dry soil versus time.

Sequencing of the 16S rRNA gene and data analysis

The reverse primers (907F) from a certain soil sample were tagged with a six-base barcode. Samples with the same barcode were separated into different pools. All the amplifications were performed in a 50 μL reaction mixture containing 10 ng of DNA, 0.2 μM of each primer and 25 μL of Premix Ex Taq Version 2.0 (Takara). Amplifications were run with the following conditions: 94°C for 3 min; 30 cycles of 94°C for 30 s, 58°C for 1 min and 72°C for 1 min; 72°C for 10 min. PCR products were verified via electrophoresis on a 1 % agarose gel. The expected fragments were excised from the gel and purified with the Universal DNA Purification Kit (TIANGEN, China). The concentrations of the purified fragments were then confirmed using the QuantiFluor dsDNA System (Promega, CA, USA). The barcoded PCR products were mixed in equal amount and mixture was then precipitated by ethanol with sodium acetate and dissolved in 50 μL sterilized Milli-Q water. The final DNA mixture was sequenced on an Illumina MiSeq PE300 platform.

QIIME (version 1.9.1) was used to analyze the sequencing data. Raw sequences were demultiplexed and low quality or ambiguous reads were removed using the default parameters in QIIME. In total, there were 4,608,293 sequences. Chimeric reads were checked and removed with "identify_chimeric_seqs.py" method using the "usearch61" option with de-novo and reference based chimera checking. Filtered sequences were then analyzed by the "pick_de_novo_otu.py" method by default. Sequences were first clustered into operational taxonomic units (OTUs) at 97% similarity level and the representative sequence for each OTU was selected. The representative sequences were then assigned to taxonomy using RDP retrained with Greengenes release (gg_13_8_otus). The sequences were clustered into 276,554 OTUs assigned with certain taxa. After filtering the OTUs with a single sequence or with an assignment with mitochondria and chloroplast, 75,658 OTUs were obtained for further analysis. After filtering, the minimum number of sequences per sample was 38,890, which was then used as the depth for sample rarefactions before calculating diversities. Alpha diversity (Shannon-Wiener index, chao1, and PD whole tree) was calculated to assess the internal

complexity of the microbial communities. The diversity between samples and groups was compared using rarefaction curves. Beta diversity was determined based on the weighted and unweighted Unifrac matrices to observe the differences in overall community compositions using PCoA analysis. Adonis was employed with 999 permutations to investigate the variation significance between different groups of microbial communities based on the Unifrac metric. Correlation analysis was conducted between values of PCoA axis 1 and abundances of the taxa to determine the taxa driving community differences. LDA Effect Size (LEfSe) based on the relative abundances of the microbial taxa was calculated to identify the corresponding taxa with higher abundance in urban, suburban, and agricultural samples. Analysis of LEfSe was performed according to the instructions on the website (http://huttenhower.sph.harvard.edu/galaxy).

Statistical analysis

Detrended Correspondence Analysis (DCA) was performed and CCA was chosen as the constrained ordination model according to the results of DCA. Significance of the environmental factors were tested by envfit function with 999 Monte Carlo permutations and variance inflation factors were calculated for each variable by vif.cca. Only factors that were confirmed to be significant (p<0.05) by envfit and were with inflation factors below 20 were accepted for the CCA analysis. The overall significance of CCA and each axis were tested by analysis of variance (ANOVA) like permutation tests using anova.cca function. The 18 factors were divided into two sets (soil chemicals and urban data). The explanatory proportions were calculated by running partial CCA using one set against the other. Then these 18 variables were used for the Mantel tests. They were also fitted to the PCoA ordinations and the significant ones (p<0.05) were kept in the plots. All these analyses including ANOSIM and PERMANOVA were performed using the *vegan* package, while the phylum heatmap was generated using the *pheatmap* package.

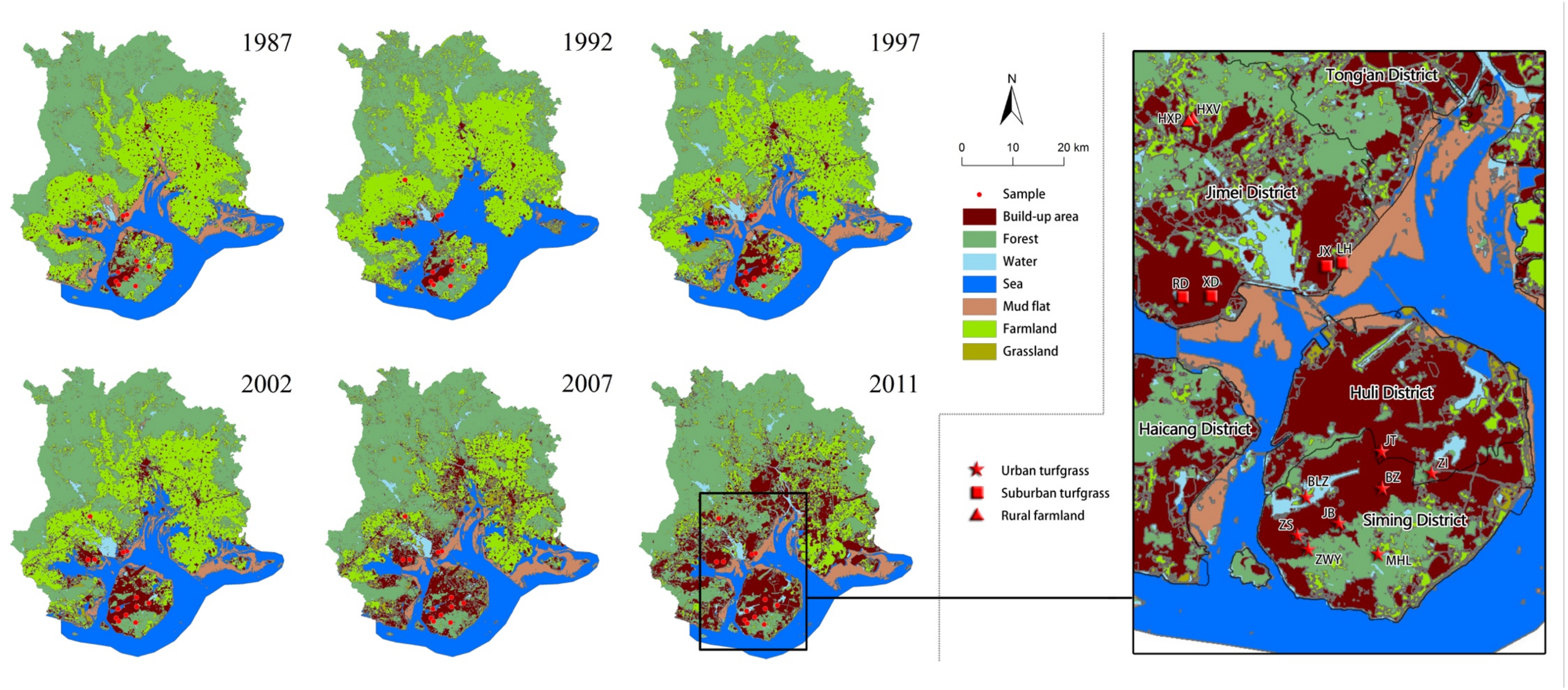

Fig. S1. Changes of land uses from 1987 to 2011 in Xiamen City and the distribution of sampling sites. Land-use data were obtained from images taken by satellite Landsat 5 (http://landsatlook.usgs.gov/viewer.html); clear satellite images from 1987, 1992, 1997, 2002, 2007 and 2011 were selected to extract the data set[1-6]. The base map was made by ArcGIS Desktop Version 10.3 (Environmental Systems Research Institute, CA, USA).

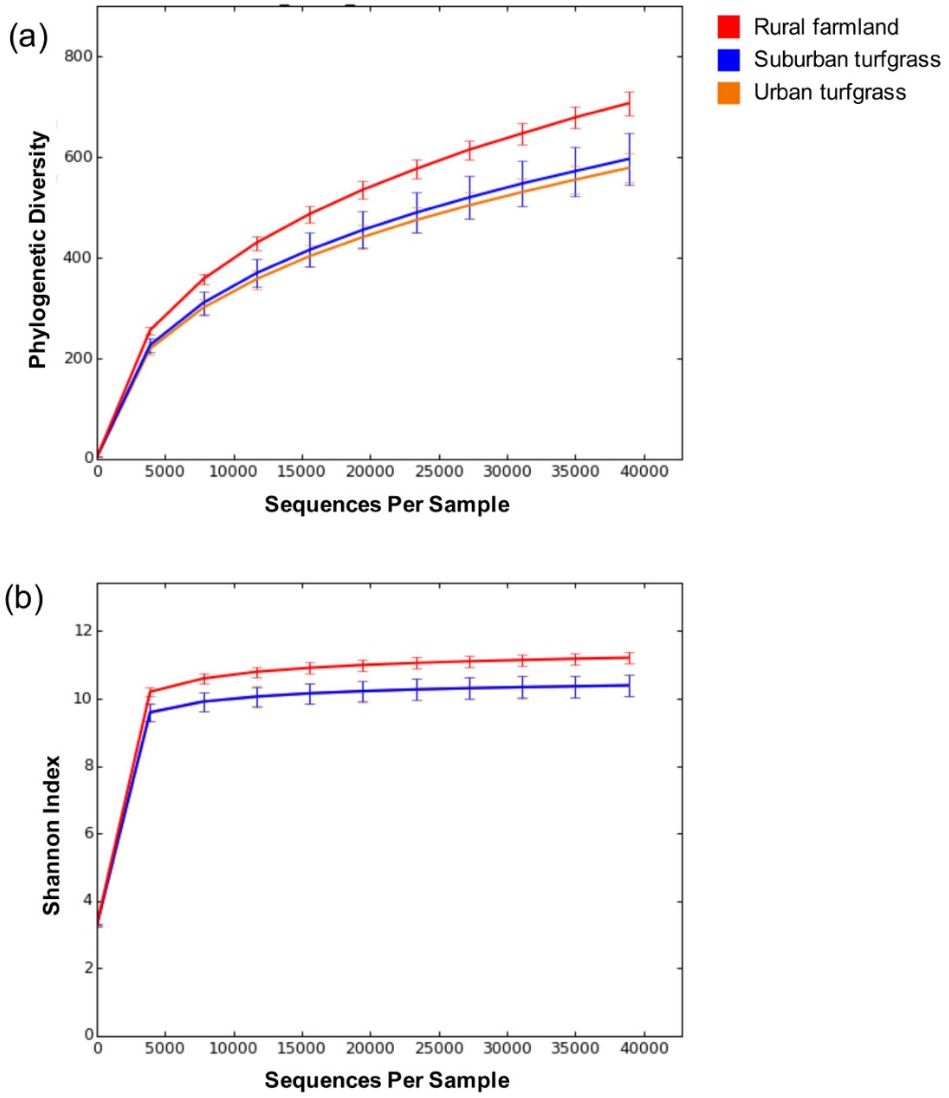

Fig. S2 Rarefaction curves of the microbial communities from the urban, suburban and rural agricultural soils based on the phylogenetic diversity (a) and Shannon index (b).

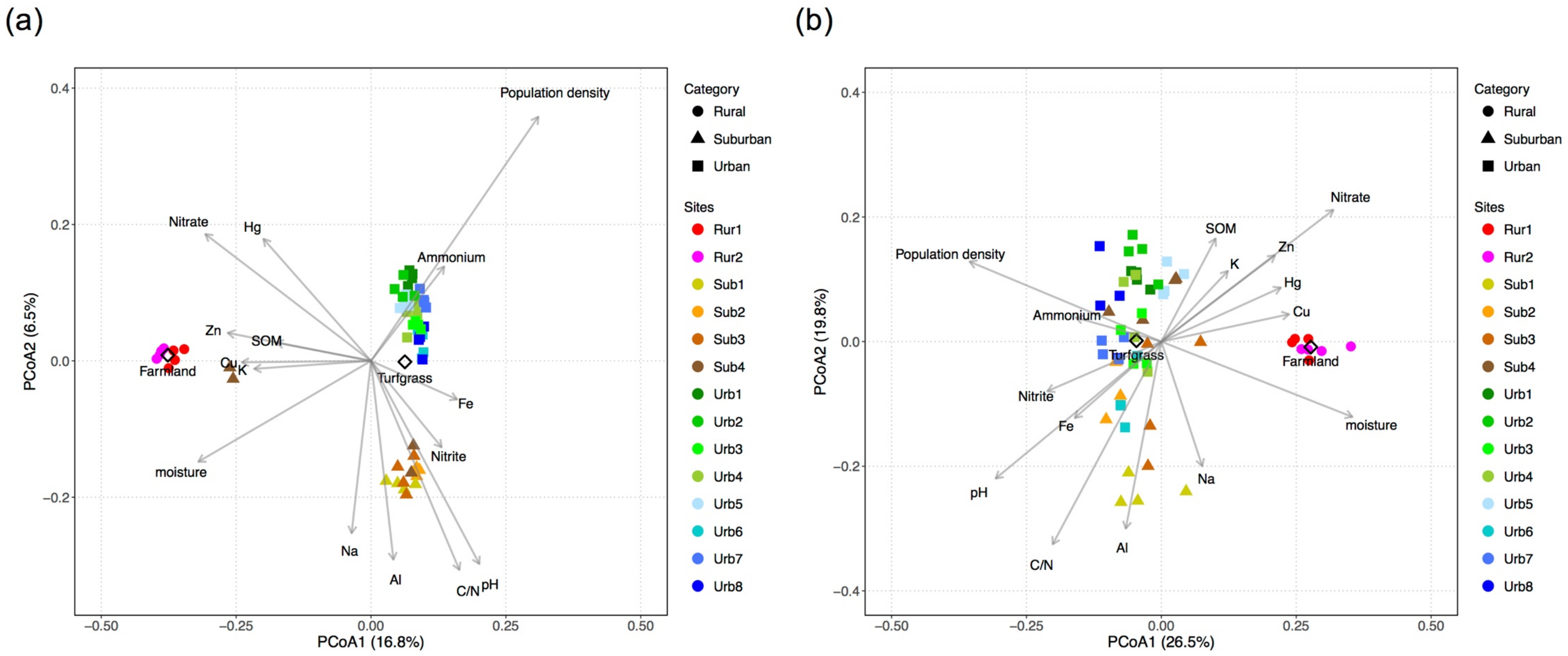

Fig. S3 PCoA plots of the microbial communities from urban, suburban and rural farmland soils based on unweighted (a) and weighted (b) Unifrac distances. The main ordination shows similarity between samples and the correlation between environmental variables and ordination axes. The two centroids representing "Turfgrass" and "Farmland" of the land use categorical variable are shown as unfilled diamonds.

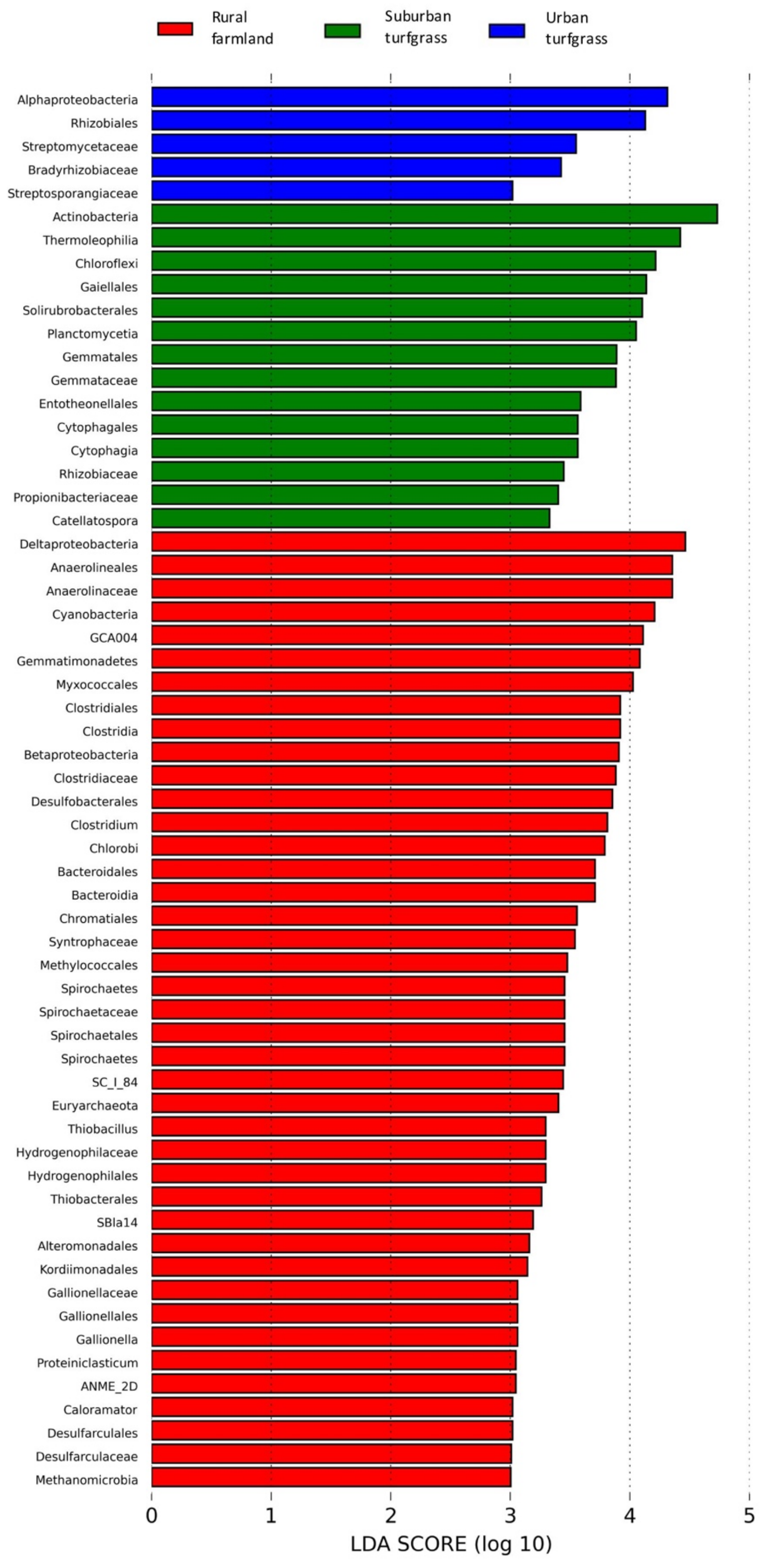

Fig. S4 Histogram of the LDA scores calculated for taxa differently abundant in urban, suburban and rural agricultural groups.

Table S1 The statistic data of Siming, Huli and Jimei in Xiamen in 2010, 2012 and 2014

Year	District	Land area (km^2)	Population (thousand)	Population density (thousand km^{-2})
2010	Siming	75.31	933	12.39
	Huli	65.78	942	14.32
	Jimei	255.9	587	2.29
2012	Siming	75.31	959	12.73
	Huli	65.78	971	14.76
	Jimei	255.9	607	2.37
2014	Siming	75.31	983	13.05
	Huli	65.78	1006	15.29
	Jimei	255.9	633	2.47

The population is the permanent residential population.

Table S2 Soil chemical properties in each site

Sites	Moisture ($g\ g^{-1}$)	pH	TC (%)	TN (%)	C/N	SOM	NO_2^- ($mg\ kg^{-1}$)	NO_3^- ($mg\ kg^{-1}$)	NH_4^+ ($mg\ kg^{-1}$)
Urb1	0.03	6.23	0.83	0.07	12.75	1.46	0.61	14.43	1.17
Urb2	0.13	6.40	1.20	0.09	13.73	2.12	0.53	49.47	0.30
Urb3	0.23	7.05	1.10	0.07	17.24	2.25	1.29	9.06	0.41
Urb4	0.19	7.20	1.13	0.08	14.57	2.09	1.70	64.65	0.58
Urb5	0.09	5.94	0.94	0.08	11.05	1.75	0.29	69.36	ND
Urb6	0.14	7.21	0.72	0.05	14.48	1.37	1.56	9.37	0.61
Urb7	0.11	6.16	0.65	0.05	12.61	1.15	0.58	13.20	0.95
Urb8	0.12	6.74	2.32	0.19	12.44	4.39	0.65	74.08	ND
Sub1	0.15	7.29	0.60	0.03	20.77	1.18	1.08	1.25	0.20
Sub2	0.15	7.64	1.22	0.08	15.83	2.28	1.47	16.16	0.58
Sub3	0.18	6.97	0.68	0.05	14.85	1.34	0.49	9.14	0.23
Sub4	0.15	6.96	2.02	0.15	13.68	3.40	1.28	39.06	0.24
Rur1	0.25	5.54	1.83	0.19	9.86	3.36	0.20	134.96	ND
Rur2	0.38	5.70	1.55	0.14	11.38	2.72	0.30	109.01	ND
$LSD_{0.05}$	0.04	0.52	0.61	0.04	1.90	1.16	0.81	34.3	0.68
$LSD_{0.01}$	0.06	0.69	0.82	0.06	2.53	1.54	1.07	45.6	0.91

Values are given as the mean for each site. $LSD_{0.05}$ and $LSD_{0.01}$ indicate that the least significant differences are accepted at $p<0.05$ and $p<0.01$ level, respectively, when the difference of the mean exceeds the LSD. ND, not detected.

Table S3 Concentrations of elements in different sampling sites (mg kg^{-1} dw soil)

Sites	K	Ca	Na	Mg	Cu	Mn	P	Zn	Cr	Ba	Fe	Al	Ni	As	Hg	Pb
Urb1	2010.9	975.0	211.1	832.1	4.1	222.0	174.9	26.9	1.6	35.7	8764.3	12549.9	1.7	3.2	0.8	18.0
Urb2	3950.0	2022.1	294.1	1415.6	20.5	476.9	335.3	65.7	9.2	80.4	12518.7	41586.8	7.7	4.6	0.8	59.9
Urb3	2261.5	1467.5	331.7	878.1	26.6	258.3	328.4	47.7	19.7	50.1	53849.9	106950.6	16.9	6.7	0.1	35.7
Urb4	2070.3	3825.4	313.3	928.2	18.9	271.0	303.8	43.1	21.8	66.5	40983.1	90164.0	10.6	6.1	0.8	25.6
Urb5	2013.7	7853.2	287.7	4903.5	9.8	579.2	438.6	47.7	14.4	57.7	24406.1	30240.2	14.2	3.0	0.2	20.8
Urb6	2014.1	1452.9	261.6	868.6	30.2	339.7	258.7	59.9	19.2	63.7	44386.8	101697.2	13.8	3.7	0.6	30.7
Urb7	4442.1	832.4	267.1	802.7	10.2	178.4	171.5	36.0	19.9	88.0	29093.2	71692.6	8.7	6.3	0.6	34.1
Urb8	5069.4	3366.3	467.5	2674.5	38.5	547.9	666.1	111.2	34.2	174.5	37397.1	81116.8	19.3	6.3	0.3	46.1
Sub1	2439.9	2140.0	403.5	980.5	7.1	204.0	149.9	19.8	0.9	53.9	22786.8	100985.3	11.7	4.5	0.2	30.9
Sub2	4369.1	3518.4	312.5	1165.6	22.1	408.3	310.4	58.0	9.0	83.4	33371.1	96992.9	9.1	7.0	0.0	67.1
Sub3	871.8	1009.9	608.1	769.2	20.8	1236.5	191.3	46.3	15.5	160.0	30105.1	106586.2	19.6	1.2	0.0	86.6
Sub4	5780.5	12800.7	270.1	1429.0	14.9	249.0	222.5	43.7	3.3	63.0	24774.8	76607.4	5.0	4.4	0.3	41.5
Rur1	5506.4	2751.4	437.6	2104.7	32.6	494.2	1632.0	90.2	16.2	122.2	25293.8	87622.8	13.8	7.1	2.5	51.0
Rur2	4530.0	1857.8	343.8	1377.8	55.5	533.9	1684.4	89.3	22.0	108.3	14544.8	48585.9	21.8	3.9	0.7	46.1
$LSD_{0.05}$	837.6	5160.9	87.6	442.4	14.1	161.0	149.6	20.4	12.2	25.6	7553.6	49700.5	9.1	2.7	0.7	16.7
$LSD_{0.01}$	1114.0	6864.0	116.5	588.4	18.8	214.1	199.0	27.1	16.2	34.1	10046.3	66101.7	12.1	3.7	1.0	22.2

Values are given as the mean for each site. $LSD_{0.05}$ and $LSD_{0.01}$ indicate that the least significant differences are accepted at $p<0.05$ and $p<0.01$ level, respectively, when the difference of the mean exceeds the LSD. dw, dry weight.

Table S4 Soil texture of each soil in different sampling sites

	Clay (%)	Silt (%)	Sand (%)	Soil type
Urb1-1	0	22.707853	77.292143	Loamy Sand
Urb1-2	0	26.519733	73.480266	Loamy Sand
Urb1-3	0	26.839377	73.160625	Loamy Sand
Urb1-4	0	20.717072	79.282931	Loamy Sand
Urb2-1	0	65.422675	34.577327	Silt loam
Urb2-2	0	66.227579	33.772422	Silt loam
Urb2-3	0	69.731238	30.268764	Silt loam
Urb2-4	0	58.926453	41.073546	Silt loam
Urb3-1	0	87.636217	12.363784	Silt soil
Urb3-2	0	86.622924	13.377076	Silt soil
Urb3-3	0	78.789652	21.210346	Silt loam
Urb3-4	0	85.030531	14.969468	Silt soil
Urb4-1	3.583115	46.267973	50.148912	Sandy loam
Urb4-2	1.437366	40.069477	58.493156	Sandy loam
Urb4-3	0.34615	40.191482	59.462365	Sandy loam
Urb4-4	1.157335	48.328466	50.514201	Sandy loam
Urb5-1	0	71.298437	28.701564	Silt loam
Urb5-2	0	53.203007	46.79699	Sandy loam
Urb5-3	0	61.680655	38.319347	Silt loam
Urb5-4	0	60.79981	39.200191	Silt loam
Urb6-1	0	76.769698	23.230301	Silt loam
Urb6-2	0	72.564803	27.435195	Silt loam
Urb6-3	0	77.441833	22.558165	Silt loam
Urb6-4	0	87.175764	12.824237	Silt soil
Urb7-1	0	73.118784	26.881215	Silt loam
Urb7-2	0	74.288232	25.711767	Silt loam
Urb7-3	0	79.128445	20.871555	Silt loam
Urb7-4	0	81.521665	18.478336	Silt loam
Urb8-1	0	59.770236	40.229761	Silt loam
Urb8-2	0	70.752003	29.247999	Silt loam
Urb8-3	0	71.870517	28.129485	Silt loam
Urb8-4	0	62.221473	37.778525	Silt loam
Sub1-1	0	74.056323	25.94368	Silt loam
Sub1-2	0	79.289993	20.710009	Silt loam
Sub1-3	0	69.42648	30.573518	Silt loam
Sub1-4	0	72.611234	27.388767	Silt loam
Sub2-1	0	82.16052	17.839479	Silt loam
Sub2-2	0	81.120603	18.879396	Silt loam
Sub2-3	0	90.471332	9.528669	Silt soil

Sub2-4	0	74.483555	25.516448	Silt loam
Sub3-1	0	79.718327	20.281673	Silt loam
Sub3-2	0	78.89905	21.100951	Silt loam
Sub3-3	0	79.286736	20.713265	Silt loam
Sub3-4	0	79.134943	20.865056	Silt loam
Sub4-1	0	76.270771	23.729227	Silt loam
Sub4-2	0	89.062516	10.937484	Silt soil
Sub4-3	0	76.263455	23.736542	Silt loam
Sub4-4	0	69.927348	30.072653	Silt loam
Rul1-1	0	86.498835	13.501164	Silt soil
Rul1-2	0	79.048566	20.951431	Silt loam
Rul1-3	0	86.411975	13.588025	Silt soil
Rul1-4	0	81.387704	18.612296	Silt loam
Rul2-1	0	64.137744	35.862256	Silt loam
Rul2-2	2.412694	67.807484	29.779824	Silt loam
Rul2-3	0	64.744036	35.255963	Silt loam
Rul2-4	0	76.382004	23.617998	Silt loam

Table S5 Potential N_2O emission, denitrification activities and nitrification rate in different sampling sites

Sites	Potential N_2O emission ($\mu g\ N_2O\ g^{-1}$ dw h^{-1})	PDA ($\mu g\ N_2O\ g^{-1}$ dw h^{-1})	$N_2O/(N_2O+N_2)$ Ratio	PNR (mg $NO_3^-\ kg^{-1}$ dw h^{-1})
Urb1	0.42	0.47	0.90	0.42
Urb2	0.10	1.24	0.81	1.01
Urb3	0.69	1.42	0.49	0.31
Urb4	0.82	1.65	0.50	2.00
Urb5	0.45	0.57	0.82	0.84
Urb6	0.47	0.85	0.56	0.28
Urb7	0.31	0.34	0.94	0.19
Urb8	1.68	2.49	0.69	2.52
Sub1	0.75	0.75	0.48	0.15
Sub2	0.59	0.90	0.64	0.18
Sub3	0.62	0.82	0.72	0.14
Sub4	1.21	2.20	0.60	1.08
Rur1	1.61	2.05	0.80	0.64
Rur2	2.62	4.10	0.62	2.36
$LSD_{0.05}$	0.65	0.95	0.17	0.79
$LSD_{0.01}$	0.86	1.27	0.23	1.05

Values are give as mean for each site. $LSD_{0.05}$ and $LSD_{0.01}$ indicate that the least significant differences are accepted at $p<0.05$ and $p<0.01$ level, respectively, when the difference of the mean exceeds the LSD. dw, dry weight.

Table S6 Absolute abundances (copies g^{-1} dry weight soil), ratios and relative abundances of genes involved in nitrification and denitrification in different samples

Sites	Bacterial 16S (10^{10})	Archaeal 16S (10^{8})	AOA (10^{7})	AOB (10^{6})	AOA/ AOB	*nirK* (10^{8})	*nirS* (10^{8})	*nirK*/ *nirS*	*nosZ* I (10^{7})	*nosZ* II (10^{7})	*nosZ* I/ *nosZ* II	*nir*/ *nos*	AOA/ 16S	AOB/ 16S (10^{-4})	*nirK*/ 16S (10^{-2})	*nirS*/ 16S (10^{-2})	*nosZ* I/ 16S (10^{-3})	*nosZ* II/ 16S (10^{-3})
Urb1	1.21	5.10	23.8	2.69	95.5	1.52	1.32	1.16	2.57	3.78	0.72	4.52	0.48	2.25	1.26	1.10	2.13	0.31
Urb2	2.32	7.69	34.2	1.82	383.6	2.68	5.21	0.57	4.27	16.2	0.28	4.04	0.44	0.85	1.17	2.19	1.84	0.72
Urb3	0.73	3.88	16.0	0.88	222.3	1.87	4.57	0.43	2.10	3.13	0.70	13.3	0.58	1.22	2.57	6.33	2.87	0.42
Urb4	1.91	6.31	17.1	4.22	34.7	1.75	3.73	0.52	2.94	32.2	0.14	2.03	0.28	2.25	0.94	2.18	1.63	1.73
Urb5	1.35	5.56	18.9	8.69	27.1	1.67	0.95	1.76	2.40	16.9	0.15	1.39	0.34	6.24	1.30	0.74	1.78	1.43
Urb6	0.35	0.45	2.52	0.39	60.4	0.36	0.20	1.94	0.66	4.14	0.27	1.69	0.56	1.04	1.10	0.58	1.93	1.20
Urb7	0.91	3.67	27.8	1.24	242.6	1.50	0.40	3.72	1.23	2.33	0.57	6.62	0.65	1.35	1.68	0.45	1.33	0.25
Urb8	1.92	7.26	8.34	4.36	19.0	3.95	0.80	5.23	4.33	6.02	0.73	4.88	0.12	2.33	2.13	0.46	2.28	0.31
Sub1	0.32	0.14	0.47	0.11	43.3	0.44	2.16	0.22	1.65	1.37	1.26	9.20	0.21	0.41	1.43	7.88	4.80	0.53
Sub2	1.88	1.86	2.44	0.17	177.1	2.87	0.79	3.77	2.47	13.7	0.17	2.30	0.16	0.10	1.54	0.45	1.28	0.75
Sub3	0.57	1.49	0.21	0.21	11.7	0.73	9.24	0.09	2.61	4.71	0.74	14.7	0.01	0.45	1.26	14.8	4.71	0.89
Sub4	2.44	23.0	8.16	35.1	2.90	3.89	9.96	0.39	7.72	3.67	2.25	12.7	0.04	14.2	1.61	4.11	3.17	0.16
Rur1	3.25	15.6	3.89	13.4	3.14	3.30	21.8	0.16	11.0	14.5	0.85	11.0	0.03	4.14	1.02	6.75	3.38	0.44
Rur2	3.49	10.2	4.32	48.6	1.03	4.82	21.5	0.23	12.3	20.7	0.62	8.22	0.04	14.8	1.72	7.38	4.09	0.72
$LSD_{0.05}$	0.70	2.43	9.93	10.5	101.4	0.79	3.94	0.97	1.80	7.12	0.56	4.84	0.20	2.82	0.61	3.19	1.80	0.68
$LSD_{0.01}$	0.94	3.24	13.2	14.0	134.9	1.05	5.24	1.30	2.39	9.47	0.74	6.43	0.26	3.74	0.81	4.25	2.40	0.91

Values are given as the mean for each site. $LSD_{0.05}$ and $LSD_{0.01}$ indicate that the least significant differences are accepted at $p<0.05$ and $p<0.01$ level, respectively, when the difference of the mean exceeds the LSD. dw, dry weight.

Table S7 Pairwise ANOSIM and PERMANOVA between urban, suburban and rural based on the unweighted and weighted Unifrac distances

	ANOSIM				PERMANOVA			
	Unweighted		Weighted		Unweighted		Weighted	
	R	Significance	R	Significance	R^2	*p*	R^2	*p*
Global test	0.829	0.001***	0.643	0.001***	0.209	0.001***	0.325	0.001***
Urban vs. Suburban	0.842	0.001***	0.357	0.001***	0.262	0.001***	0.170	0.001***
Urban vs. Rural	1.000	0.001***	0.992	0.001***	0.840	0.001***	0.623	0.001***
Suburban vs. Rural	0.953	0.001***	0.769	0.001***	0.763	0.001***	0.544	0.001***

*, significance level at 0.05; **, significance level at 0.01; ***, significance level at 0.001.

Table S8 Mantel tests between environmental variables and microbial community based on unweighted and weighted Unifrac distances

Variables	Unweighted		Weighted	
	r	*p*	r	*p*
Moisture	0.055	0.533	-0.046	0.581
pH	0.176	0.001***	0.245	0.001***
C/N	0.124	0.116	0.440	0.001***
SOM	0.164	0.038*	0.216	0.035*
NO_2^-	-0.019	0.741	0.015	0.844
NO_3^-	0.061	0.434	0.233	0.007**
NH_4^+	-0.016	0.823	-0.113	0.151
K	0.132	0.048*	0.142	0.030*
Na	0.101	0.230	0.196	0.046*
Cu	-0.046	0.54	0.165	0.052
Ba	0.052	0.542	0.281	0.003**
Fe	0.019	0.807	-0.012	0.841
Al	0.214	0.006**	0.188	0.005**
Zn	-0.029	0.710	0.236	0.012*
Hg	0.217	0.006**	0.085	0.090
Pb	0.135	0.079	0.161	0.047*
Population density	0.572	0.001***	0.301	0.001***

*, significance level at 0.05; **, significance level at 0.01; ***, significance level at 0.001.

Table S9 Primers and conditions used to target and quantify the 16S rRNA and functional genes

Gene Name	Primers (5'→ 3')	qPCR Condition	Reference
Archaea 16S rRNA gene	arch21F (TTCCGGTTGATCCYGCCGGA)	95°C for 2 min; 40 cycles of 95°C for 10 s, 59°C for	Ref. 7
	arch958R (YCCGGCGTTGAMTCCAATT)	40 s and 72°C for 40 s (recording)	Ref. 7
Bacteria 16S rRNA gene	515F (GTGCCAGCMGCCGCGG)	95°C for 5 min; 40 cycles of 95°C for 30 s, 58°C for	Ref. 8
	907R (CCGTCAATTCMTTTRAGTTT)	30 s and 72°C for 1 min (recording)	Ref. 9
Archaeal *amoA*	amoAF (STAATGGTCTGGCTTAGACG)	94°C for 8 min; 40 cycles of 94°C for 45 s, 61.5°C for	Ref. 10
	amoAR (GCGGCCATCCATCTGTATGT)	1 min, and 72°C for 1 min (recording)	Ref. 10
Bacterial *amoA*	amoA1F (GGGGTTTCTACTGGTGGT)	94°C for 3 min; 40 cycles of 94°C for 30 s, 55°C for	Ref. 11
	amoA2R (CCCCTCKGSAAAGCCTTCTTC)	30 s, and 72°C for 45 s (recording)	Ref. 11
nirK	F1aCu (TCATGGTSCTGCCGCG)	94°C for 2 min; 40 cycles of 94°C for 30 s, 57°C for 1	Ref. 12
	R3Cu (GCCTCGATCAGRTTGTGGTT)	min, and 72°C for 1 min (recording)	Ref. 12
nirS	cd3aF (GTSAACGTSAAGGARACSGG)	94°C for 2 min; 40 cycles of 94°C for 30 s, 57°C for 1	Ref. 13
	R3cd (GASTTCGGRTGSGTCTTGA)	min, and 72°C for 1 min (recording)	Ref. 14
nosZ I	nosZ-F (CGYTGTTCMTCGACAGCCAG)	95°C for 1 min; 95°C for 15 s, 61.5°C for 15 s, and	Ref. 15
	nosZ-1622R (CGSACCTTSTTGCCSTYGCG)	72°C for 34 s (recording)	Ref. 14
nosZ II	nosZ-II-F (CTIGGICCIYTKCAYAC)	95°C for 2 min; 40 cycles of 95°C for 15 s, 54°C for	Ref. 16
	nosZ-II-R (GCIGAICARAAITCBGTRC)	30 s, 72°C for 30 s and 80°C for 30 s (recording)	Ref. 16

Table S10 Primers used to amplify V4 and V5 regions of 16S rRNA gene

Name	Barcode	Sequence (5’ → 3’)
515F	-	GTGCCAGCMGCCGCGG
907R	-	CCGTCAATTCMTTTRAGTTT
907-R1	ATCACG	CCGTCAATTCMTTTRAGTTT
907-R2	CGATGT	CCGTCAATTCMTTTRAGTTT
907-R3	TTAGGC	CCGTCAATTCMTTTRAGTTT
907-R4	TGACCA	CCGTCAATTCMTTTRAGTTT
907-R5	ACAGTG	CCGTCAATTCMTTTRAGTTT
907-R6	GCCAAT	CCGTCAATTCMTTTRAGTTT
907-R7	CAGATC	CCGTCAATTCMTTTRAGTTT
907-R8	ACTTGA	CCGTCAATTCMTTTRAGTTT
907-R9	GATCAG	CCGTCAATTCMTTTRAGTTT
907-R10	TAGCTT	CCGTCAATTCMTTTRAGTTT
907-R11	GGCTAC	CCGTCAATTCMTTTRAGTTT
907-R12	CTTGTA	CCGTCAATTCMTTTRAGTTT
907-R13	AGTCAA	CCGTCAATTCMTTTRAGTTT
907-R14	AGTTCC	CCGTCAATTCMTTTRAGTTT
907-R15	ATGTCA	CCGTCAATTCMTTTRAGTTT
907-R16	CCGTCC	CCGTCAATTCMTTTRAGTTT
907-R17	GTAGAG	CCGTCAATTCMTTTRAGTTT
907-R18	GTCCGC	CCGTCAATTCMTTTRAGTTT

References

1 NASA Landsat Program, 1987, Landsat TM scene LT51190431987257BJC00, L1G, USGS, Sioux Falls, 09/14/1987.

2 NASA Landsat Program, 1992, Landsat TM scene LT51190431992175BJC00, L1G, USGS, Sioux Falls, 06/23/1992.

3 NASA Landsat Program, 1997, Landsat TM scene LT51200431997275BJC00, L1G, USGS, Sioux Falls, 10/02/1997.

4 NASA Landsat Program, 2002, Landsat TM scene LT51190432002314BJC00, L1G, USGS, Sioux Falls, 11/10/2002.

5 NASA Landsat Program, 2007, Landsat TM scene LT51190432007008BJC00, L1G, USGS, Sioux Falls, 01/08/2007.

6 NASA Landsat Program, 2011, Landsat TM scene LT51200432011106BJC00, L1G, USGS, Sioux Falls, 04/16/2011.

7 DeLong, E. F. Archaea in coastal marine environments. *P. Natl. Acad. Sci. USA*. **89**, 5685-5689 (1992).

8 Miteva, V. I., Sheridan, P. P. & Brenchley, J. E. Phylogenetic and Physiological Diversity of Microorganisms Isolated from a Deep Greenland Glacier Ice Core. *Appl. Environ. Microbiol.* **70**, 202-213 (2004).

9 Reysenbach, A.-L., Longnecker, K. & Kirshtein, J. Novel bacterial and archaeal lineages from an in situ growth chamber deployed at a Mid-Atlantic Ridge hydrothermal vent. *Appl. Environ. Microbiol.* **66**, 3798-3806 (2000).

10 Francis, C. A., Roberts, K. J., Beman, J. M., Santoro, A. E. & Oakley, B. B. Ubiquity and diversity of ammonia-oxidizing archaea in water columns and sediments of the ocean. *P. Natl. Acad. Sci. USA.* **102**, 14683-14688 (2005).

11 Rotthauwe, J. H., Witzel, K. P. & Liesack, W. The ammonia monooxygenase structural gene amoA as a functional marker: molecular fine-scale analysis of natural ammonia-oxidizing populations. *Appl. Environ. Microbiol.* **63**, 4704-4712 (1997).

12 Hallin, S. & Lindgren, P. E. PCR detection of genes encoding nitrite reductase in denitrifying bacteria. *Appl. Environ. Microbiol.* **65**, 1652-1657 (1999).

13 Michotey, V., Méjean, V. & Bonin, P. Comparison of methods for quantification of cytochrome cd 1-denitrifying bacteria in environmental marine samples. *Appl. Environ. Microbiol.* **66**, 1564-1571 (2000).

14 Throbäck, I. N., Enwall, K., Jarvis, Å. & Hallin, S. Reassessing PCR primers targeting *nirS*, *nirK* and *nosZ* genes for community surveys of denitrifying bacteria with DGGE. *FEMS Microbiol. Ecol.* **49**, 401-417 (2004).

15 Kloos, K., Mergel, A., Rösch, C. & Bothe, H. Denitrification within the genus *Azospirillum* and other associative bacteria. *Funct. Plant Biol.* **28**, 991-998 (2001).

16 Jones, C. M., Graf, D. R., Bru, D., Philippot, L. & Hallin, S. The unaccounted yet abundant nitrous oxide-reducing microbial community: a potential nitrous oxide sink. *ISME J.* **7**, 417-426 (2013).
